# Selective Cytotoxicity of Goniothalamin against Hepatoblastoma HepG2 Cells

**DOI:** 10.3390/molecules16042944

**Published:** 2011-04-06

**Authors:** Mothanna Al-Qubaisi, Rosli Rozita, Swee-Keong Yeap, Abdul-Rahman Omar, Abdul-Manaf Ali, Noorjahan B. Alitheen

**Affiliations:** 1 Department of Cell and Molecular Biology, Faculty of Biotechnology and Biomolecular Sciences, University Putra Malaysia (UPM), 43400 Serdang, Selangor, Malaysia; 2 Department of Obstetric and Gynaecology, Faculty of Medicine and Health Sciences, University Putra Malaysia (UPM), 43400 Serdang, Selangor, Malaysia; 3 Department of Veterinary Pathology and Microbiology, Faculty of Veterinary Medicine, University Putra Malaysia (UPM), 43400 Serdang, Selangor, Malaysia; 4 Faculty of Agriculture and Biotechnology, University Sultan Zainal Abidin (UniSZA), Kampus Kota, Jalan Sultan Mahmud, 20400 Kuala Terengganu, Malaysia

**Keywords:** goniothalamin, HepG2 cell, liver Chang cell, cytotoxicity

## Abstract

Liver cancer has become one of the major types of cancer with high mortality and liver cancer is not responsive to the current cytotoxic agents used in chemotherapy. The purpose of this study was to examine the *in vitro* cytotoxicity of goniothalamin on human hepatoblastoma HepG2 cells and normal liver Chang cells. The cytotoxicity of goniothalamin against HepG2 and liver Chang cell was tested using MTT cell viability assay, LDH leakage assay, cell cycle flow cytometry PI analysis, BrdU proliferation ELISA assay and trypan blue dye exclusion assay. Goniothalamin selectively inhibited HepG2 cells [IC_50_ = 4.6 (±0.23) µM in the MTT assay; IC_50_ = 5.20 (±0.01) µM for LDH assay at 72 hours], with less sensitivity in Chang cells [IC_50_ = 35.0 (±0.09) µM for MTT assay; IC_50_ = 32.5 (±0.04) µM for LDH assay at 72 hours]. In the trypan blue dye exclusion assay, the Viability Indexes were 52 ± 1.73% for HepG2 cells and 62 ± 4.36% for Chang cells at IC_50_ after 72 hours. Cytotoxicity of goniothalamin was related to inhibition of DNA synthesis, as revealed by the reduction of BrdU incorporation. At 72 hours, the lowest concentration of goniothalamin (2.3 µL) retained 97.6% of normal liver Chang cells proliferation while it reduced HepG2 cell proliferation to 19.8% as compared to control. Besides, goniothalamin caused accumulation of hypodiploid apoptosis and different degree of G2/M arrested as shown in cell cycle analysis by flow cytometry. Goniothalamin selectively killed liver cancer cell through suppression of proliferation and induction of apoptosis. These results suggest that goniothalamin shows potential cytotoxicity against hepatoblastoma HepG2 cells.

## 1. Introduction 

Liver cancer is one of the leading causes of worldwide cancer mortality, with an estimated 1 million deaths annually and 5-year survival rates of less than 5% [1]. Liver cancer incidence of 4 to 15 per 100,000 has been reported in Western countries, as compared with 120 per 100,000 in Asia and Africa [2]. Previous research has revealed that liver cancer is largely refractory to chemotherapy because of tumor heterogeneity and the development of multidrug resistance phenotypes [3]. Thus, up to now, availability of treatments for liver cancer remains unsatisfactory [4] because liver cancer cells are present p53 gene mutations and tend to be more aggressive and extremely resist to chemotherapy [5].

Doxorubicin is one of the best drugs for systemic chemotherapy which works well with a variety of other chemotherapy agents, including epirubicin, mitoxantrone, cisplatin and etoposide [6]. It is often used in patients with liver cancer disseminated beyond the liver, although the response rates are generally only 15%. Moreover, doxorubicin is expensive and carries serious side effects ranging from nausea, vomiting, mucositis, ulceration and necrosis of the colon to acute myeloid leukemia with a preleukemic phase and heart failure [7].

**Figure 1:**
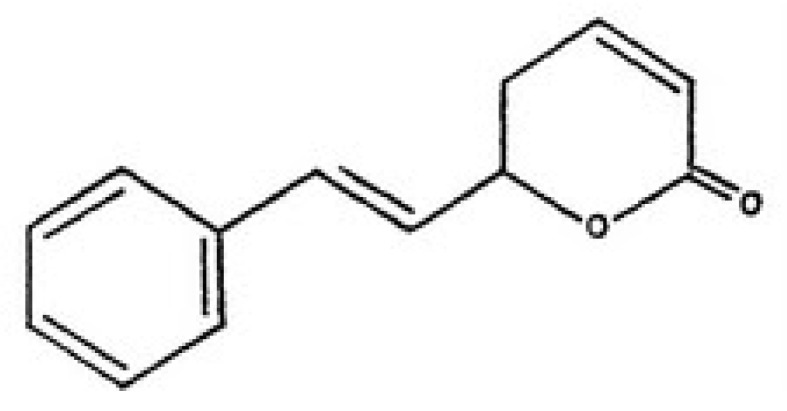
Structure of goniothalamin.

Plant bioactive compounds are normally cheaper and cause fewer side effects with respect to chemotherapy. Thus, the search for drugs (or compounds) extracted from plant as potential cytotoxic agents for hepatoblastoma is an important line of research in the discovery of novel anticancer candidates. Goniothalamin (Figure 1) is a styryl-lactone compound isolated from the root and stem of *Goniothalamus macrophyllus* [8]. The cytotoxicity and antimicrobial activity of goniothalamin isolates had been evaluated by many researchers. Goniothalamin showed promising cytotoxicity against colon cancer cell line, breast cancer cell lines, lung carcinoma and others [9,10,11]. In addition, goniothalamin showed more selective cytotoxic activity against cancer cell lines than normal cell lines [9]. 

To our knowledge, no specific study had been reported addressing the selective cytotoxic effects of goniothalamin on the hepatoblastoma HepG2 and normal Chang cell lines. Thus, in this present study, we have evaluated the *in vitro* cytoxicity of goniothalamin and doxorubicin (as a positive control) on the hepatoblastoma HepG2 and normal Chang cell lines using different assays. Results from both cancerous and normal cell were then compared to determine the selective activity.

## 2. Results and Discussion 

### 2.1. Results

**Figure 2:**
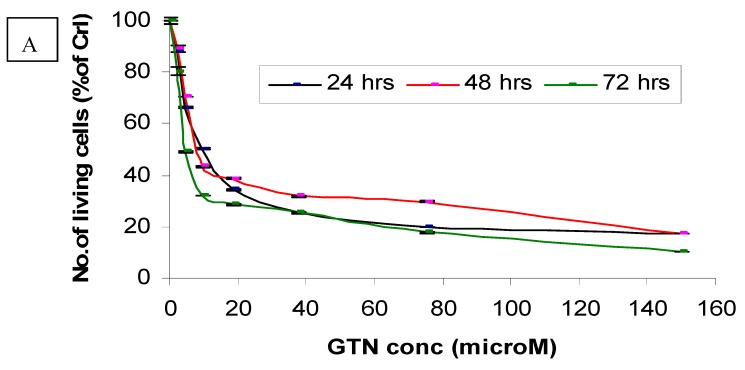

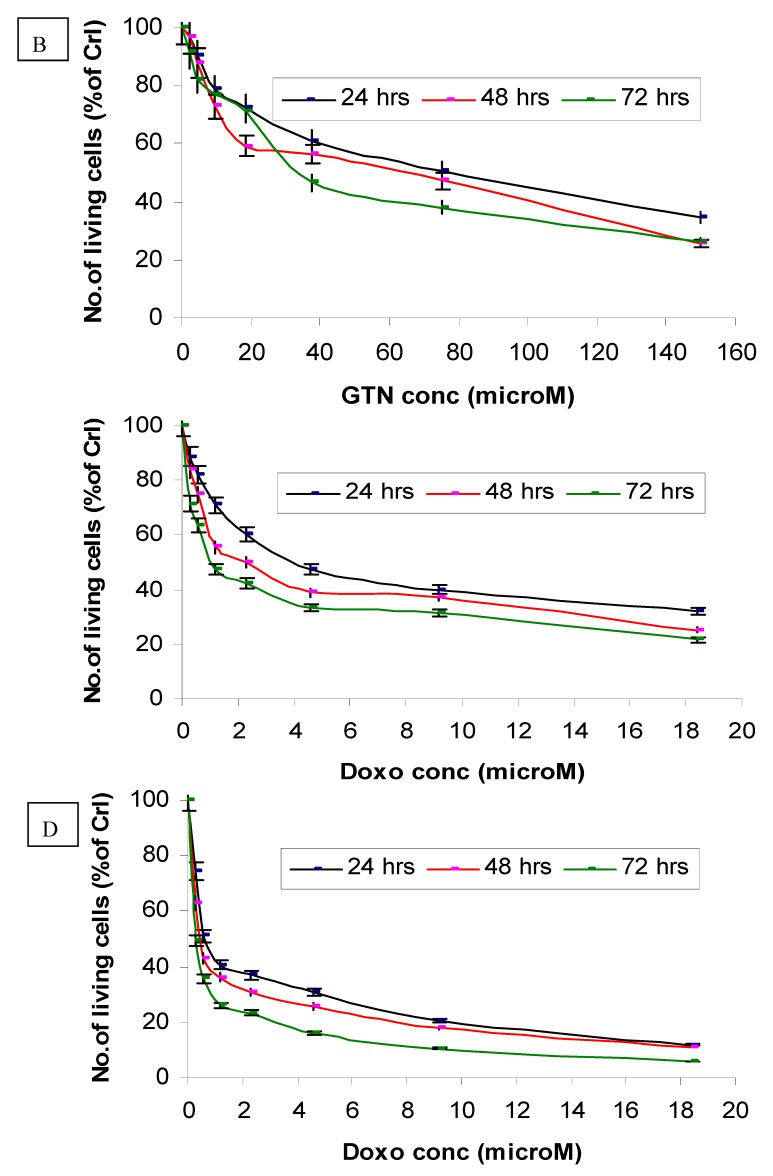
Effects of goniothalamin treatment in HepG2 (A) and Chang cells (B) and doxorubicin treatment in HepG2 (C) and Chang cells (D). Twenty-four hours after seeding of cells in 96 well plates, goniothalamin and doxorubicin were added to the final concentrations shown in the figure. At 24 h (●), 48 h (■) and 72 h (♦) of treatment, effects of goniothalamin and doxorubicin against the viability of treated cells were evaluated through mitochondrial activity using the MTT assay.

Cytotoxicity of goniothalamin on HepG2 and Chang cells was assessed using a MTT assay. Responses of HepG2 cells toward increase concentrations of goniothalamin were exponential. HepG2 cells experienced a significant decrease in viability at low concentrations of goniothalamin, with an eventual decline at the highest concentrations tested. The estimated IC_50_ values of goniothalamin (concentration causing death of 50% of HepG2 cells) ranged between 9.4 and 2.3 μM (Figure 2A) after 72 hours. 

The *in vitro* dosage- and time-dependent effects of goniothalamin against cultured normal human (Chang) liver cells are shown in Figure 2B. Following incubation of these cells with 75 μM of goniothalamin, approximately 49%, 53% and 63% decreases of cell growth were observed after 24, 48 and 72 hours of incubation, respectively, as compared to the untreated control cells. Under the same conditions, incubation of these cells with 37.5 μM of goniothalamin, resulted in 39%, 44% and 54% decreases of cell growth at these same time points (Figure 2B).

The selectivity index (SI) of HepG2 and Chang liver cells obtained using IC_50_ values are shown in Table 1. The SI of goniothalamin for Chang liver cells was 7.6 times higher than that for HepG2 cells. Furthermore, compared with goniothalamin, doxorubicin (positive control) showed higher cytotoxicity towards normal liver Chang cell line.

Measurement of LDH activity is another indicator of cell viability through evaluation of the cell membrane permeability. The enzyme activity is measured externally, as it leaks from dead cells which lose their membrane integrity. LDH leakage detection is based on the loss of NADH due to its oxidation to NAD^+^, resulting in the conversion of pyruvate to lactate. Untreated cells should retain LDH in their cells and have minimal loss over the time of the assay. Triton X-100-treated cells that give the maximum loss of LDH were used as the positive control in this assay.

**Figure 3:**
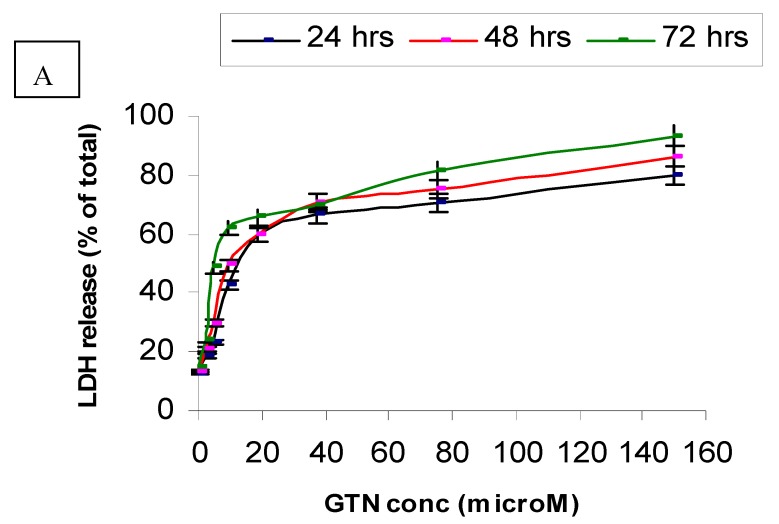

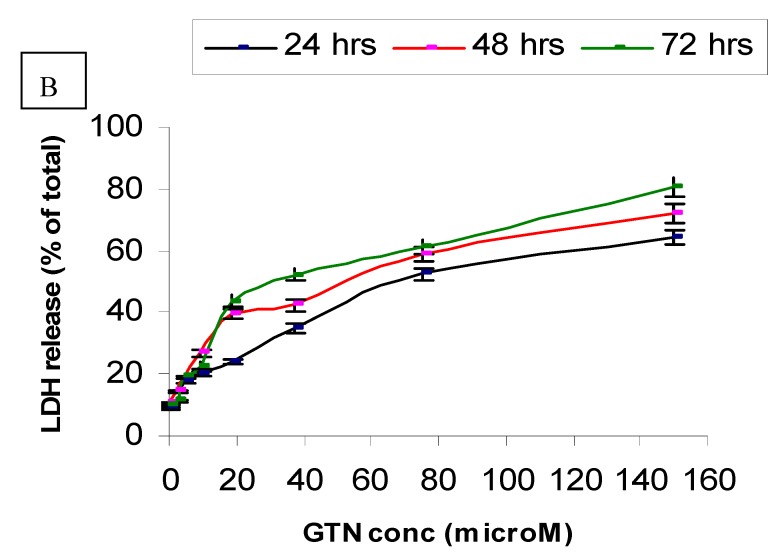
LDH leakage in HepG2 (A) and Chang cells (B) treated with goniothalamin. Cells (1 × 10^4^) with different concentrations of goniothalamin were incubated for the indicated times. The cytotoxicity was expressed as percentage LDH release as compared to the maximum release of LDH from Triton-X100-treated cells.

Figure 3 and Table 2 show the leakage of LDH in the culture media after treatment with goniothalamin for 24, 48 and 72 hours, respectively. Exposure of HepG2 to goniothalamin (9.4 µM) raised LDH leakage from 40% up to 62 % after 72 hours. Doubling the goniothalamin concentration from 37.5 to 150 µM further increased the leakage of LDH from 60% to 80%, 70% to 86% and 70% to 93%, after incubation for 24, 48 and 72 hours, respectively. After 72 hours of incubation with goniothalamin, 4.6 µM was enough to increase the death percentage up to 50%, as compared to Triton X-100-treated cells (maximum release of LDH) (Figure 3A). 

On the other hand, Chang cells were less sensitive to goniothalamin as compared to HepG2. In Chang cells, only a small increase (19%) in LDH leakage was found in the cells treated with 4.7 µM of the compound after 72 hours of incubation. At the concentrations of 2.3, 4.7 or 9.4 µM, no significant toxicity caused by goniothalamin in Chang cell line was observed on completion of the time of incubation. At a concentration of 37.5 µM, goniothalamin did not cause more than 52% loss of viability of Chang cells (Figure 3B). When HepG2 and Chang cells were treated with doxorubicin, LDH was released in a time and concentration dependent manner. Besides, Chang cells were more sensitive to doxorubicin (data not shown) and less sensitive to goniothalamin.

The ability of a substance to affect specific phases of the cell cycle may provide clues as to its mechanism(s) of action. To determine the effects of goniothalamin on the cell cycle, HepG2 cells were treated with IC_50_ levels of goniothalamin (determined from the MTT assay) for 24, 48 and 72 hours, respectively. The cells were stained with PI and the DNA contents analyzed by flow cytometry. Untreated HepG2 cells showed a normal cell cycle distribution, with approximately 81.2% in G_0_/G_1_ phase; 6.8% in S phase and 12.0% in G_2_/M phase. Decrease of cells in G_0_/G_1_ was noted when compared to untreated control (Table 3). Moreover, a sub-G_1_ apoptotic peak was induced by goniothalamin in a time-dependent manner. After 72 hours, goniothalamin at the IC_50_ (4.63 µM) increased the sub-G_1_ apoptotic fraction up to 59%.

The effect of goniothalamin on the cell cycle distribution of non-malignant Chang cells was also assessed. Goniothalamin showed a time-dependent effect on the cell cycle of Chang cells. Accordingly, goniothalamin induced a marked apoptosis in a time-dependent manner at IC_50_ concentrations. As expected, the increase in the apoptotic population was associated with a concomitant enlargement of the pre-phase population, as observed in cell cycle analysis. The number of S-phase cells in the Chang cell cultures was significantly decreased after 72 hours (Table 3).

For Chang cells treated with IC_50_ amounts of doxorubicin, reciprocal induction in the number of cells in S and M/G_2_ phase was observed in a time independent manner. Moreover, doxorubicin induced a sub-G_1_ peak five-fold greater than untreated control cells after 72 hours exposure (Table 3).

To further confirm the potential of goniothalamin-induced cell death HepG2 cells, a BrdU cell proliferation assay was performed. The cell proliferation percentage was measured by the incorporation of the thymidine analogue bromodeoxyuridine into DNA. In untreated HepG2, the absorbances (OD) at 450 nm wavelength were 1.445, 2.188 and 2.553 for 24, 48 and 72 hours, respectively. The cell proliferation rate was reduced in a concentration dependent manner after the addition of goniothalamin, compared to untreated control cells after all the times (Figure 3). The anti-proliferative effects of goniothalamin at IC_50_ concentrations against HepG2 were noticed at all the time points, that is, OD value had declined from 0.635 at 24 hours to 0.372 after 72 hours. The lowest concentration showed a slight decline in the curve after 24 hours of incubation, but after 72 hours the curve decline sharply to produce a low OD value of 0.505 as compared to untreated cells. Inhibition of DNA synthesis at the highest concentration (150 µM) is also time-dependent and is the most severe. Doxorubicin only had higher inhibition effect than goniothalamin at IC_50_ concentration for 24 hours.

**Figure 4:**
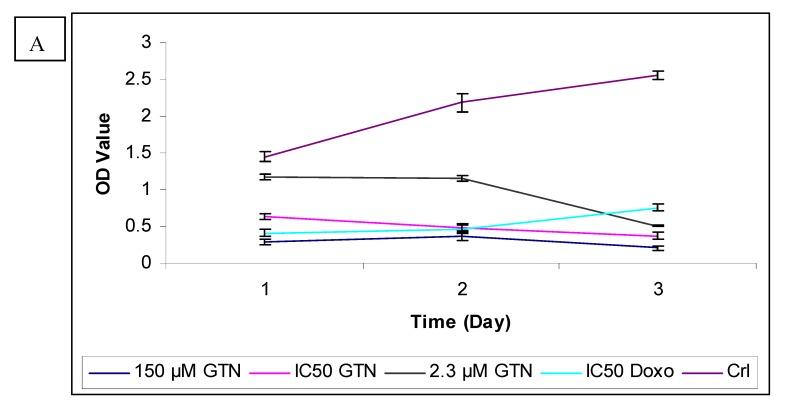

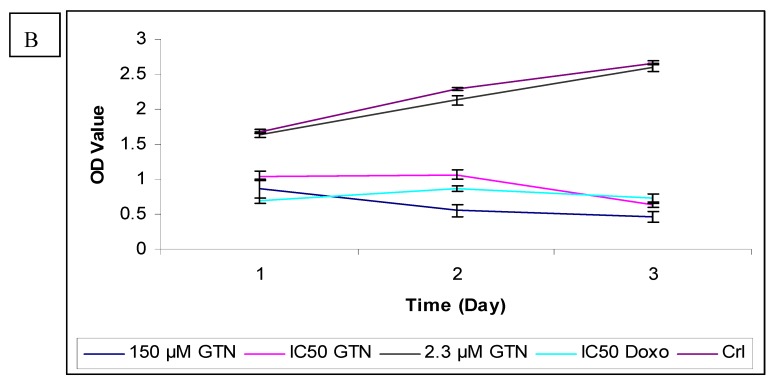
Effects of goniothalamin and doxorubicin on the proliferation of HepG2 (A) and Chang (B) cells *in vitro*. Goniothalamin inhibits cell proliferation in a time- and dose-dependent manner. After treatment with 2.3 µM (IC_50_ based on MTT assay results) and 150 µM of goniothalamin and IC_50_ of doxorubicin (as positive control) for 24, 48 and 72 hours, cellular proliferation of HepG2 and Chang cells was assayed using BrdU incorporation ELISA.

The inhibition of goniothalamin on cellular DNA synthesis in Chang cells was also measured by BrdU incorporation. Exposure of Chang cells to goniothalamin (IC_50_) for 24 and 72 hours resulted in a reduction in percentage of cells incorporating BrdU from 62.4% to 23.5%, in comparison with untreated cells (Figure 4). 

**Figure 5:**
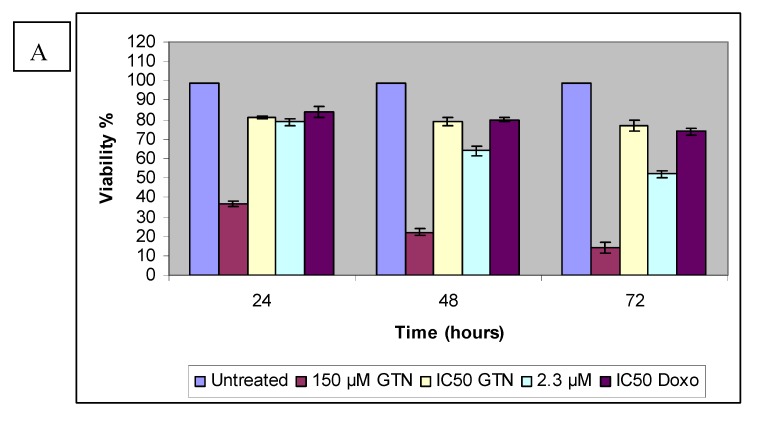

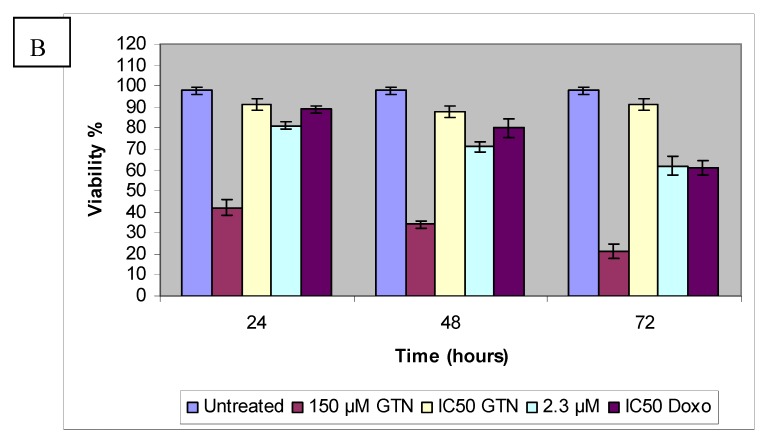
HepG2 (A) Chang (B) cells were treated with goniothalamin (GTN) and at the indicated concentration and doxorubicin IC_50_ (Doxo) for 24, 48 and 72 hours. The viable cell number was assessed by cell counting in a trypan blue assay; Control values were set as 100%.

In contrast to HepG2, incubation for 3 days with the lowest concentration of goniothalamin did not show a significant reduction in proliferation of Chang cells. The highest concentration caused a significantly strong time-dependent decreased of BrdU incorporation. Like Hep2 cells, doxorubicin-treated Chang cells showed lower proliferation than the IC_50_ of goniothalamin at 24 hours. As supported by MTT and LDH results, goniothalamin inhibited cell proliferation towards HepG2 in a time- and dose-dependent manner as shown in the BrdU incorporation ELISA method. The effects of goniothalamin on HepG2 and Chang cell growth were also analyzed by viable cell counts using trypan blue dye exclusion assay for 24, 48 and 72 hours after treatment with different doses of goniothalamin. As the results indicated, cell growth was significantly reduced after exposure to goniothalamin and this reduction was time and dose dependent. The ratio between viable cell numbers in goniothalamin-treated cells and control cells was reported as the Viablity Index (VI, Figure 5A and Figure 5B). 

### 2.2. Discussion

Many researchers are now interested in examining the use of herbal medicines as a health care method [12]. Herbal medicines continue to be accepted forms of treatment in the Orient, and the plant-derived drugs based on traditional practices represent a huge proportion of the pharmaceutical production in modern Western countries [13]. Development of biologically targeted agents that exploit differences between cancerous and normal cells and permit greater specificity for cancer cells with less damage to normal cells is still the ultimate goal in the field of antineoplastic drug discovery [14]. 

Until now, no ideal cytotoxicity assay has been developed; hence, it is always advisable to support results with more assays where possible. Besides, it is important to compare the cytotoxicity of a novel compound between cell lines and even with other commercial cytotoxic agents.

In this study, we showed that goniothalamin (at IC_50_ concentrations) was cytotoxic against HepG2 cell lines, but less toxic on Chang cells after 72 hours of exposure. The highest concentration of goniothalamin (150 µM) showed the highest toxicity on both cell lines. Based on a MTT test, doxorubicin treatment resulted in concentration- and time-dependent toxicities on both HepG2 and Chang cells. The cytotoxic effects of the lowest concentration of doxorubicin (0.29 µM) on HepG2 and Chang cells were evident at 24 hours of incubation. However, the sensitivity of these HepG2 and Chang cells to goniothalamin and doxurubicin was considerably different. These results are similar to those of a previous study that showed the cytotoxicity of a styrylpyrone derivative that was apparently selective for both human breast cancer cell lines MCF-7 and MDA-MB-231, but had no significant toxicity towards two non-malignant cells normal human liver cell lines like CCL 13 and normal bovine kidney MDBK [15].

Cell cycle arrest is a common feature of cells that are undergoing terminal differentiation and defective proliferation. Based on the growth inhibitory effect (as shown in MTT results) and loss of membrane integrity (as shown in LDH release into the medium) caused by goniothalamin on HepG2 cell lines, the cell cycle progression in response to goniothalamin was investigated. Table 3 further indicated that the cytotoxicity caused by goniothalamin might be derived from its potency in inducing marked apoptotic cell death after 24 hours. Cell cycle arrest induced by goniothalamin had been previously reported in MDA-MB-231 human breast cancer cells [10]. Nevertheless, after 72 hours incubation, apoptotic cell death was the main contributor to toxicity, while the accumulation in the G_2_/M phase was decreased, as compared to 24 hours. In contrast, doxorubicin induced less apoptotic cell death (Table 3) than goniothalamin after 72 hours. Our results confirmed that goniothalamin had higher cytotoxicity than doxorubicin via inducing marked apoptotic cell death in hepatoma cells. Doxorubicin is classified as an antitumor antibiotic, made from natural products produced by species of the soil fungus *Streptomyces* [7]. It acts during multiple phases of the cell cycle and the cytotoxic effect of doxorubicin are generally considered to be cell-cycle specific where accumulation in G_2_/M phase can normally be detected in doxorubicin treated HepG2 [16]. In some liver cancer cells, G_2_/M arrest occurred soon after doxorubicin treatment [17], and significant apoptosis was observed subsequently, followed by p53-independent apoptosis [18]. Unlike doxorubicin, data obtained using flow cytometric analysis indicated that decreased cell mass of goniothalamin treated cells was due to a significant sub-diploid population of cells. These results suggested that goniothalamin triggered the apoptotic cell death of malignant and non-malignant liver cells. In the present study, we found that goniothalamin had higher percentage of cells with hypodiploid DNA content (subG_1_). For both cell lines, DNA histograms exhibited a prominent decrease of G_0_/G_1_ cell population after treatment with goniothalamin and doxorubicin. 

Similarly, an apoptotic peak appeared before the G_1_ phase when non-malignant Chang cells were treated with goniothalamin. This time-dependent effect were significantly apparent upon the induction of apoptosis, while cell cycle arrest was at G_2_/M. This indicated that the reduction of MTT value in goniothalamin-treated Chang cells was due to growth arrest, but not to cell death alone. In addition, the results showed that Chang cells treated with doxorubicin for 72 hours showed a stepwise increase in subG_1_ population. 

BrdU is incorporated in the DNA during the S phase of the cell cycle and is detected by a colorimetric BrdU cell proliferation ELISA [19]. From the BrdU ELISA assay results, an increase in exposure time to goniothalamin appeared to have a greater cell proliferation reducing effect in HepG2 than Chang cell lines under most circumstances. In contrast, as seen from Figure 3A, the proliferation of HepG2 was lower for goniothalamin at IC_50_ exposures than for doxorubicin at IC_50_ exposure for 72 hours. This suggested that doxorubicin can induce toxicity to HepG2 earlier than goionthalamin. However, goniothalamin became more toxic after longer incubation times. This finding is similar to the results obtained from the MTT assay, where increasing toxicity of goniothalamin was found to be associated with an increase in exposure time. Although both 24 and 48 hour assays showed large declines in HepG2 proliferation when treated with 150 µM as compared to the control group, the 72 hour assay showed a sharp decline in proliferation at the lowest concentration (2.3 µM). In contrast, the lowest concentration showed a small (2%) decrease in cell proliferation of Chang cells, compared to untreated cells after 72 hours exposure. This selectivity finding agreed with the MTT assay results.

An overall decline in BrdU incorporation in the goniothalamin-treated cells indicated the decrease occurred uniformly from the G_1_ and S regions (as explained in the results of cell cycle analysis). Our results confirmed and extended previous results concerning the effects of goniothalamin on the cell cycle in which researchers observed a decrease of BrdU incorporation in vascular smooth muscle cells (VSMCs) following goniothalamin treatment [20].

Based on the results of the trypan blue dye exclusion assay, increasing goniothalamin exposure time had a detrimental effect on HepG2 cell viability. Treatment of HepG2 cells with 9.2, 8.3 and 4.6 µM of goniothalamin resulted in a dose- and time-dependent inhibition of cell growth amounting to 79% (24 hours), 64% (48 hours), and 52% (72 hours), respectively. A similar situation may be seen in the normal liver cell line. The comparison between 24 to 72 hour exposure data showed that Chang cell viability was also decreased with increasing exposure time. Figure 4 demonstrated the effects on cell viability in Chang cells after exposure to goniothalamin or doxorubicin for 24 to 72 hours. Unlike HepG2 cells, at lowest goniothalamin concentrations, cultures with longer exposure times occasionally had measures of viability similar to those with shorter exposure times for only Chang cells. This observation was consistent with MTT and LDH results.

It appears that HepG2 and Chang cells reacted differently to goniothalamin exposure in the trypan blue dye exclusion assay. Cells count results indicated that viability of HepG2 cells was significantly inhibited by goniothalamin at 2.3 µM concentration (Figure 4). In contrast, no obvious cytotoxicity was detected in Chang cells treated with comparable doses. The HepG2 cells thus appeared to be more sensitive to goniothalamin and HepG2 viability was consistently lower than the Chang cell viability at all of the goniothalamin concentrations tested. This may be contributed to by changes in the cell membrane triggered by the high experimental goniothalamin concentrations, which may increase the uptake of trypan blue dye and the subsequent membrane alteration may have gradually resulted in cell death. Some evidence has indicated that goniothalamin exposure can alter the membrane properties [21]. This hypothesis could explain the increasing of lactate dehydrogenase (LDH) released to the media, after exposure to goniothalamin for 24, 48 and 72 hours, respectively. 

In this study, four different bioassays have shown selective targeting of a human liver tumor cell line by goniothalamin and the HepG2 and Chang cell lines responded to goniothalamin in a different manner. Compared to goniothalamin, doxorubicin evoked greater toxicity in both HepG2 and Chang cells in all assays. Nevertheless, these data provided evidence that goniothalamin fulfils two basic criteria for an effective therapeutic agent, i.e., tumor specificity and minimal toxicity towards the normal cells. 

## 3. Materials and Methods 

### 3.1. Reagents 

Goniothalamin (5-hydroxy-7-phenylhepta-2,6-dienoic acid lactone, Figure 1) was kindly provided by Prof. Dr. Abdul Manaf bin Ali [Faculty of Agriculture and Biotechnology, Universiti Darul Iman Malaysia (UDM)]. It was extracted as described previously [21]. Goniothalamin trypsin/EDTA solution was purchased from Invitrogen Co. (Carlsbad, CA, USA). Dimethylsulfoxide (DMSO), nicotinamide adenine dinucleotide (reduced form) NADH, phosphate buffered saline (PBS), Triton X-100 3-(4,5-dimethylthiazol-2-yl)-2,5-diphenyltetrazolium bromide (MTT), Dulbecco’s modified Eagle’s media (DMEM) and trypan blue dye were purchased from Sigma Chemical Company (St. Louis, MO, USA).

### 3.2. Cell lines

Two human cell lines were obtained from the American Type Culture Collection (ATCC; Rockville, MD, USA). The cell lines comprised Chang and HepG2 cells, which are characterized as hepatitis virus negative. They grow as an adherent monolayer of tightly knit epithelial cells [22].

### 3.3. MTT cytotoxicity assay

HepG2 and Chang cells were plated at 1 × 10^4^ cells/well by adding 200 μL of a 5 × 10^4^ cells/mL suspension to each well of a 96-well tissue culture plate. The plate was incubated for a sufficient time to assure attachment and 40% to 60% confluency. The media was aspirated off and replaced with fresh media (200 μL) containing goniothalamin of different concentrations (150 µM → 2.3 µM) and doxorubicin (18.4 µM → 0.29 µM). The last row was left as an untreated control. The plates were incubated at 37 °C, 5% CO_2_, for 24, 48 and 72 hours, respectively. After incubation with compounds, the media was aspirated off and replaced with fresh media. Then, MTT solution (20 μL) for a total volume of 200 μL was added in every well and incubated for 4 to 6 hours at 37 °C with 5% CO_2_. After that, MTT-containing medium was removed gently and replaced with DMSO (200 μL per well) to mix the formazan crystals until dissolved. The plates were read on microtiter plate reader at 570 nm. For each compound tested, the IC_50_ (concentration of drug needed to inhibit cell growth by 50%) is generated from the dose-response curves for each cell line.

### 3.4. Lactate dehydrogenase (LDH) assay

To determine the effect of goniothalamin on membrane permeability in HepG2 and Chang cell lines, a lactate dehydrogenase (LDH) release assay was used. The cells were seeded in 96-well culture plates at a density of 2 × 10^4^ cells/well in 100 μL volume and allowed to grow for 18 hours before treatment. After treatment, the plates were incubated for 24, 48 and 72 hours, respectively. Then, the supernatant (40 μL) was transferred to a new 96 well to determine LDH release and 6% triton X-100 (40 μL) was added to the original plate for determination of total LDH. An aliquot of 0.1 M potassium phosphate buffer (100 μL, pH 7.5) containing 4.6 mM pyruvic acid was mixed to the supernatant using repeated pipetting. Then, 0.1 M potassium phosphate buffer (100 μL, pH 7.5) containing 0.4 mg/mL reduced *β*-NADH was added to the wells. The kinetic changes were read for 1 min using ELISA microplate reader in absorbance at wavelength 340 nm. This procedure was repeated with 40 μL of the total cell lysate to determine total LDH U/well. A change of 0.001 absorbance units/min is equivalent to 1 U/L of LDH activity [23]. The percentage of LDH release was determined by dividing the LDH released into the media by the total LDH following cell lysis in the same well.

### 3.5. Cell cycle PI analysis 

The treated cells were trypsinized and washed with PBS. After centrifugation, the supernatant was removed. Then, 1 × 10^6^ cells were harvested and fixed in ice-cold ethanol (70%). The fixed cells were centrifuged at high speed (180 × *g*) for 10 minutes to adequately pellet down the cells that were suspended in the ethanol. The pellet was washed by the ice-cold PBS and then centrifuged again at 180 × *g* for 10 minutes. The final pellet was resuspended with 1 mL of RNase and incubated at 37 °C for 30 minutes. At the end of incubation, DNA binding dye PI (1 mg/mL) was added to give a final concentration of 50 µg/mL. The mixture was incubated at room temperature for 10 min in the dark. Data acquisition and analysis were performed in by flow cytometry FACS Calibur flow cytometer (Becton Dickinson, Franklin Lakes, NJ, USA) using CellQuest 3.3 software. 

### 3.6. Bromodeoxyuridine (BrdU) cell proliferation assay 

To confirm anti-proliferative effects in goniothalamin-treated cells, BrdU cell proliferation kit was used. HepG2 and Chang cells were seeded at 1.5 × 10^5^ cells/mL in a final volume of 100 μL in a 96-well culture dish and incubated overnight to allow the cells to adhere to the dish. Each sample was assayed in triplicate. Besides, the control samples include a blank (no cells) and background (cells without BrdU) was also prepared. The cells were treated with goniothalamin (and doxorubicin as positive control) for 24, 48 and 72 hours, respectively. Then, BrdU label (20 μL, diluted by 1/2,000 in media) was added to each well and incubated for 24 h at 37 °C. The media was discarded and replaced by fix/denaturing solution (200 μL). The plate was incubated for 30 min at room temperature. The fix/denaturing solution was removed by inverting the plate. Then, BrdU antibody (100 μL, diluted by 1/100 in dilution buffer) was added to each well and left for 1 hour at room temperature. The cells were washed 3 times with wash buffer. After that, secondary antibody (100 μL, anti-mouse IgG HRP conjugated) was added to each well for 30 min. The washing step was done three times with wash buffer and once with distilled water. The distilled water was removed and substrate solution (100 μL) was added to each well. The plate was left for 15 min at room temperature in the dark condition. Finally, stop solution (100 μL) was added to the wells and absorbance measured at 450–540 nm.

### 3.7. Viable cell counts using trypan blue dye exclusion assay

A half mL aliquot of cell suspension (obtained from treated and non-treated HepG2 and Chang cells), was mixed with 0.5 mL of 0.4% trypan blue dye and left for 5 min at room temperature). The cells were counted using the hemacytometer and the percentage of dead cells was determined.

## 4. Conclusions 

Goniothalamin showed potential cytotoxicity against hepatoblastoma HepG2 cells as it reduced the cell viability after 72 hours. HepG2 cells were more sensitive to goniothalamin as it has a lower IC_50_ value [IC_50_ of MTT= 4.6 (±0.23) µM; IC_50_ of LDH= 5.20 (±0.01) µM after 72 hours] as compared to normal liver Chang cells. The cytotoxicity of goniothalamin was related to the inhibition of DNA synthesis, as shown by BrdU cell proliferation and cell cycle analysis. The *in vitro* selective inhibitory effect of goniothalamin suggests the potential of this natural compound as a novel chemotherapy agent candidate for liver cancer treatment. 

## Figures and Tables

**Table 1:** The selectivity index (SI) which represents IC_50_ for normal cell line/IC_50_ for cancerous cell line after 72 hours of goniothalamin and doxorubicin treatment.

Cells	Goniothalamin IC_50_ (μM)	Doxorubicin IC_50_ (μM)
HepG2	4.63	1.06
Chang	35.01	0.28
**SI**	7.6	3.7
**IC_50-GTN_ / IC_50-Doxo_**	4.4	125.0

**Table 2:** Comparison of IC_50_ values for HepG2 and Chang cells obtained from LDH leakage assay and MTT assay following exposure to goniothalamin for 24, 48 and 72 hours.

Cell line	Duration (hours)	Cytotoxicity assay	
		IC_50_ from MTT (µM)	IC_50_ from LDH (µM)
HepG2	24	9.20	13.43
	48	8.30	8.29
	72	4.63	5.20
Chang	24	79.10	69.58
	48	63.75	55.11
	72	35.01	32.47

**Table 3:** Effects of goniothalamin (IC_50_ value) and doxorubicin (IC_50_ value) on cell cycle distribution of (a) HepG2 (b) liver Chang cells determined by flow cytometry. The cells were treated with IC_50_ levels of the compounds (taken from MTT results) for 24 hours, 48 hours, 72 hours and untreated cells and analyzed by flow cytometry. G_0_/G_1_, G_2_/M and S indicate the cell phase, and sub-G_1_ DNA content refers to the proportion of apoptotic cells.

**(a)**		**SubG_0_/G_1_ (%)**	**G_0_/G1 (%)**	**S (%)**	**G_2_+M (%)**
24h	Control	2.49 ± 0.66	81.17 ± 2.07	6.82 ± 1.07	9.52 ± 0.43
	Goniothalamin (9.2 µM)	17.44 ± 1.67	68.92 ± 1.27	7.68 ± 0.12	5.94 ± 0.22
	Doxorubicin (2.2 µM)	3.36 ± 1.02	76.46 ± 3.80	8.54 ± 0.38	11.64 ± 3.02
					
48h	Control	3.16 ± 0.81	74.53 ± 0.75	4.85 ± 1.23	17.46 ± 0.09
	Goniothalamin (8.3 µM)	46.59 ± 2.79	39.71 ± 3.50	8.03 ± 0.29	5.67 ± 4.13
	Doxorubicin (4.3 µM)	5.14 ± 2.53	65.17 ± 12.80	15.15 ± 10.96	14.54 ± 1.06
					
72h	Control	3.69± 1.93	76.81± 2.84	5.37 ± 2.07	14.13 ± 0.58
	Goniothalamin (4.6 µM)	58.83 ± 2.09	31.79 ± 5.82	7.07 ± 0.18	2.31 ± 4.19
	Doxorubicin (1.06 µM)	30.79 ± 0.57	60.64 ± 0.79	5.91 ± 0.15	2.66 ± 0.37
**(b)**		**SubG_0_/G_1_ (%)**	**G_0_/G_1_ (%)**	**S (%)**	**G_2_+M (%)**
24h	Control	4.51 ± 0.21	61.27 ± 0.94	15.62 ± 0.12	18.60 ± 0.99
	Goniothalamin (79.10 µM)	17.38 ± 7.29	55.08 ± 3.60	12.65 ± 2.79	14.89 ± 5.53
	Doxorubicin (0.83 µM)	10.71 ± 7.28	50.84 ± 6.03	22.09 ± 0.03	16.36 ± 1.28
					
48h	Control	3.74 ± 0.23	69.88 ± 1.27	10.32 ± 2.33	16.06 ± 2.09
	Goniothalamin (63.75 µM)	33.00 ± 3.40	58.68 ± 9.45	5.82 ± 0.16	2.5 ± 5.89
	Doxorubicin (0.41 µM)	11.89 ± 6.49	62.43 ± 9.80	15.31 ± 3.10	10.37 ± 0.21
					
72h	Control	4.12 ± 2.39	66.84 ± 0.81	11.50 ± 1.37	17.54 ± 1.08
	Goniothalamin (35.01 µM)	80.99 ± 0.64	11.60 ± 0.07	4.91 ± 0.13	2.5 ± 0.44
	Doxorubicin (0.28 µM)	22.06 ± 5.17	54.12 ± 1.52	15.19 ± 2.83	8.63 ± 2.17

## References

[1] El-Serag H.B., Mason A.C. (1999). Rising incidence of hepatocellular carcinoma in the United States. New. Engl. J. Med..

[2] Kubicka S., Rudolph K.L., Hanke M., Tietze M.K., Tillmann H.L., Trautwein C. (2000). Hepatocellular carcinoma in Germany: A retrospective epidemiological study from a low-endemic area. Liver.

[3] Huang C.C., Wu M.C., Xu G.W., Li D.Z., Cheng H., Tu Z.X., Jiang H.Q., Gu J.R. (1992). Overexpression of the MDR1 gene and P-glycoprotein in human hepatocellular carcinoma. J. Natl. Cancer Inst..

[4] Honda K., Sbisa E., Tullo A., Papeo P.A., Saccone C., Poole S., Pignatelli M., Mitry R.R., Ding S., Isla A., Davies A., Habib N.A. (1998). p53 mutation is a poor prognostic indicator for survival in patients with hepatocellular carcinoma undergoing surgical tumour ablation. Br. J. Cancer.

[5] Heinze T., Jonas S., Karsten A., Neuhaus P. (1999). Determination of the oncogenes p53 and C-Erb B2 in the tumour cytosols of advanced hepatocellular carcinoma (HCC) and correlation to survival time. Anticancer Res..

[6] Shah S.R., Riordan S.M., Karani J., Williams R. (1998). Tumor ablation and hepatic decompensation rates in multi–agent chemoembolization of hepatocellular carcinoma. QJM.

[7] Mehta D.K. (2006). British National Formulary.

[8] Sam T.W., Sew-Yeu C., Matsjeh S., Gan E.K., Razak D., Mohamed A.L. (1987). Goniothalamin oxide: An embryotoxic compound from *Goniothalamus macrophyllus* (Annonaceae). Tetrahedron Lett..

[9] Chatchai W., Boonsong W., Puttachat S., Arunporn I., Niwat K. (2005). Goniothalamin, a cytotoxic compound, isolated from *Goniothalamus macrophyllus* (Blume) Hook. f. & Thomson var. macrophyllus. Songklanakarin. J. Sci. Technol..

[10] Chen W.Y., Wu C.C., Lan Y.H., Chang F.R., Teng C.M., Wu Y.C. (2005). Goniothalamin induces cell cycle-specific apoptosis by modulating the redox status in MDA-MB-231 cells. Eur. J. Pharmacol..

[11] De Fátima A., Kohn L.K., Antônio M.A., de Carvalho J.E., Pilli R.A. (2005). (R)- Goniothalamin: Total syntheses and cytotoxic activity against cancer cell lines. Bioorg. Med. Chem..

[12] Newall C.A., Anderson L.A., Phillipson J.D. (1996). Herbal Medicines. A Guide for Health-care Professionals.

[13] Schulz V., Rudolf H., Tyler V.E. (1998). Rational Phytotherapy. A Physician’s Guide to Herbal Medicine.

[14] Adams J. (2001). Proteasome inhibition in cancer: Development of PS-341. Sem. Oncol..

[15] Pihie A.H., Stanslas J., Din L.B. (1998). Non-steroid receptor mediated antiproliferative activity of styrylpyrone derivative (SPD) in human breast cancer cell lines. Anticancer Res..

[16] Andreas P.S., Kerstin M., Patricia G., Gesine B., Kirsten V., Michael H., Antje K., Bernhard H., Harald S., Rajan S., Detlef S., Martin Z., Hans S. (2004). Peripheral benzodiazepine receptor ligands induce apoptosis and cell cycle arrest in human hepatocellular carcinoma cells and enhance chemosensitivity to paclitaxel, docetaxel, doxorubicin and the Bcl-2 inhibitor HA14-1. J. Hepatol..

[17] Wahl A.F., Donaldson K.L., Fairchild C., Lee F.Y., Foster S.A., Demers G.W., Galloway D.A. (1996). Loss of normal p53 function confers sensitization to Taxol by increasing G2/M arrest and apoptosis. Nat. Med..

[18] Lee T.K., Lau T.C., Ng I.O. (2002). Doxorubicin-induced apoptosis and chemosensitivity in hepatoma cell lines. Cancer Chemother. Pharmacol..

[19] Gratzner H.G. (1982). Monoclonal antibody to 5-bromo- and 5- Iododeoxyuridine: A new reagent for detection of DNA replication. Science.

[20] Chan K.M., Rajab N.F., Ishak M.H.A., Ali A.M., Yusoff K., Din L.B., Inayat-Hussain S.H. (2006). Goniothalamin induces apoptosis in vascular smooth muscle cells. Chem. Biol. Interact..

[21] Inayat-Hussain S.H., Annuar B.O., Din L.B., Ali A.M., Ross D. (2003). Loss of mitochondrial transmembrane potential and caspase-9 activation during apoptosis induced by the novel styryl-lactone goniothalamin in HL-60 leukemia cells. Toxicol. In Vitro.

[22] Tay N., Chan S.H., Ren E.C. (1990). Detection of integrated hepatitis B virus DNA in hepatocellular carcinoma cell lines by nonradioactive in situ hybridization. J. Med. Virol..

[23] Jemmerson R., LaPlante B., Treeful A. (2002). Release of intact, monomeric cytochrome c from apoptotic and necrotic cells. Cell Death Differ..

